# Vulga-Vaginal Anus

**Published:** 1895-12

**Authors:** 


VULGA-VAGINAL ANUS.—Freeman (*Medical News*, Sept. 1895.) reports a successful plastic operation for the relief of this deformity in a child twelve months old. The artificial anus opened into the vagina, just within the vulva, on the posterior wall. He made an incision over the dimple where the normal anus should be and carefully dissected around the lower end of the rectum, loosening it from the adjacent tissue and the vagina, and after bringing the gut down, it was carefully sutured to the skin incision, The fistula leading into the vagina was dissected out and the vaginal wound closed by sutures. The after results were perfect, so far as could be judged from so young a patient, in which the absolute state of fæcal continence could not be positive. He also quotes Curling as having collected statistics of eleven cases, ten of which were successfully operated upon, and one terminated fatally.

I have reported one case successfully operated upon—about a year ago.—*Chicago Medical Recorder*, April 5, 1895.

